# Role of tumor microenvironment in cancer progression and therapeutic strategy

**DOI:** 10.1002/cam4.5698

**Published:** 2023-02-21

**Authors:** Qingjing Wang, Xueting Shao, Yuxuan Zhang, Miaojin Zhu, Frederick X. C. Wang, Jianjian Mu, Jiaxuan Li, Hangping Yao, Keda Chen

**Affiliations:** ^1^ Shulan International Medical College Zhejiang Shuren University Hangzhou China; ^2^ Institute of Pharmaceutical Biotechnology & Research Center for Clinical Pharmacy, The First Affiliated Hospital, School of Medicine Zhejiang University Hangzhou China; ^3^ State Key Laboratory for Diagnosis and Treatment of Infectious Diseases, National Clinical Research Center for Infectious Diseases, The First Affiliated Hospital, School of Medicine Zhejiang University Hangzhou China; ^4^ The EnMed Program at Houston Methodist Hospital Texas A&M University College of Medicine and College of Engineering Houston Texas USA

**Keywords:** cancer immunotherapy, cancer progression, PD‐1, PD‐L1, tumor microenvironment

## Abstract

Cancer is now considered a tumor microenvironment (TME) disease, although it was originally thought to be a cell and gene expression disorder. Over the past 20 years, significant advances have been made in understanding the complexity of the TME and its impact on responses to various anticancer therapies, including immunotherapies. Cancer immunotherapy can recognize and kill cancer cells by regulating the body's immune system. It has achieved good therapeutic effects in various solid tumors and hematological malignancies. Recently, blocking of programmed death‐1 (PD‐1), programmed death‐1 ligand‐1 (PD‐L1), and programmed death Ligand‐2 (PD‐L2), the construction of antigen chimeric T cells (CAR‐T) and tumor vaccines have become popular immunotherapies Tumorigenesis, progression, and metastasis are closely related to TME. Therefore, we review the characteristics of various cells and molecules in the TME, the interaction between PD‐1 and TME, and promising cancer immunotherapy therapeutics.

## Graphical abstract

In the past decades, the treatment methods for tumors have been changing rapidly. Traditional treatment methods, such as radiotherapy and chemotherapy regimens, have gradually revealed their shortcomings. As scientists continue to study the tumor microenvironment, new cellular properties, and immune checkpoints regarding TME are being discovered, and immunotherapy based on monoclonal antibodies, engineered cells, tumor vaccines, and other technologies have shown good therapeutic effects and lower risks. This review presents the latest research about TME and the latest progress in immunotherapy.

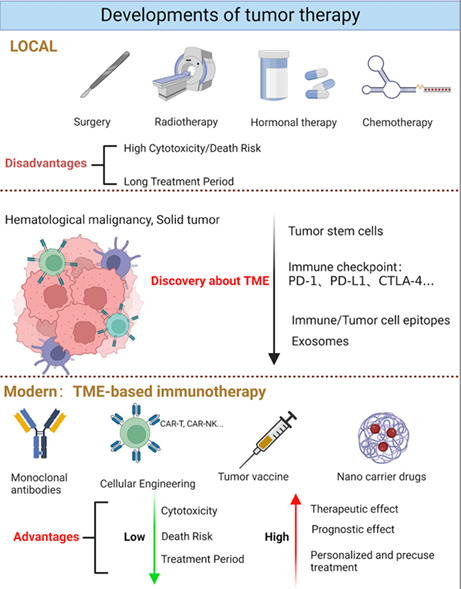

## 1 INTRODUCTION

Cancer is ranked as one of the five most serious diseases in the world, and its incidence rate is increasing year by year. Cancer has the biological characteristics of abnormal cell differentiation and proliferation, and uncontrolled growth, invasion, and metastasis.^1^ Surgical treatment, chemotherapy, and radiotherapy are the most widely used cancer treatment methods, among which chemotherapy and radiotherapy are used more frequently; however, they suffer from drug resistance and serious side effects, and their curative effects remain poor.^1^, ^2^ In recent years, various cells and molecules that contribute to tumor development in the TME have been proven to also affect cancer immunotherapy.^3^, ^4^ Tumor survival and function are regulated or promoted by the tumor microenvironment (TME). The interaction between TME structural components and cells permits cancer cells to acquire an invasive phenotype, spreading to distant sites from the primary site via a complex and multistep metastatic cascade. Tumor‐associated M2 macrophages promote growth and induce immunosuppression.^5^ The many exosomes produced by mesenchymal cells augment cancer cell migration.^6^ Moreover, Cancer‐associated fibroblasts (CAFs) and the tumor extracellular matrix (ECM) play a decisive role in the migration of cancer cells and cancer progression and modulate the response to therapy.^7^, ^8^ Programmed death‐1 (PD‐1) and programmed death‐1 ligand‐1 (PDL‐1), cancer immunotherapy, principally PD‐1/PD‐L1 pathway blockade, shows high feasibility.^9^ Indeed, the US Food and Drug Administration (FDA) has approved seven PD‐1/PD‐L1 pathway‐targeting monoclonal antibodies.^10^ In the present review, we discuss the functions of different structural and cellular components of tumors that regulate metastatic processes; targeted strategies inhibiting tumor invasion employing microRNAs (miRNAs), modified exosomes, nanoparticles, and small molecule inhibitors; and current and future strategies to reshape the TME with the aim of halting harmful metastatic processes, thereby improving therapeutic outcomes.

## 2 THE FUNCTION OF THE TME IN THE PROGRESSION AND METASTASIS OF CANCER

### 2.1 Complexity of the TME

The occurrence of a tumor is regulated by an abnormal immune response and changes in homeostasis.^11^ Cancer growth and metastasis are affected by immune cell and cancer cell interactions in the TME.^12^, ^13^ The TME regulates tumor cell basic survival and promotes their function. The aggression and metastatic spread of cancer cells to distant locations are promoted by the interaction between TME cellular and structural constituents. Growing evidence indicates that innate immune cells (macrophages, neutrophils, DCs, innate lymphocytes, myeloid inhibitory cells, and NK cells) and adaptive immune cells (T cells and B cells) contribute to tumor progression when present in the TME.^14^ In particular, cytotoxic CD8+ T cells (CTLs), have a vital function in tumor prevention and elimination. However, in many patients, CTL‐mediated tumor killing ultimately fails to clear cancer cells, leading to disease progression, largely because of effector CTL progression toward depleted CTLs.^15^ Moreover, tumor metastasis also depends on the interaction of various cells and factors in the TME.

The external and internal tumor cell environments are associated closely with tumor occurrence and metastasis. This process involves tumor‐located tissue metabolism, function, and structure, and the tumor cells' internal environment (cytoplasmic and nuclear). The TME is formed after tumor cells colonize normal tissues and change the surrounding microenvironment through the recruitment of CAFs cancer‐associated fibroblasts (CAFs), immune cell regulation and regulation of their secreted factors, and the formation of neovascularization by vascular endothelial cells.^7^ In addition to immune cell components, which play a key role, the TME contains a variety of non‐immune stromal cell components, including endothelial cells, fibroblasts, and tissue‐specific cells, all of which play a large role in signature tumor events, such as angiogenesis, ECM extracellular matrix (ECM) invasion, and metastasis. Increasing evidence shows that these stromal cells have vital functions in immune escape and immune checkpoint blockade (ICB) resistance mechanisms.^16^, ^17^, ^18^ CAFs are the core components of the TME and not only interact with cancer cells, but also affect other components of the TME, such as the ECM and immune infiltration. Histopathological analysis showed that the content of CAFs is related to the prognosis of different tumors, and CAFs can also regulate the therapeutic effect.^19^ The high rate of angiogenesis in the TME, the resulting vascular system abnormalities, and high interstitial pressure within the tumor, weakens immune cell infiltration and checkpoint inhibitor penetration.^16^ Additionally, tissue‐specific stromal cells might also play a role in tumor resistance to ICBs. The tumor ECM has an important function in the progression of cancer, the migration of cancer cells, and treatment response regulation.^20^

The metabolic state of the TME is another factor that affects tumor immunogenicity through a variety of mechanisms. Hypoxic tumors show reduced expression of the type I major histocompatibility complex (MHC) on tumor cells and dendritic cells (DCs).^21^ Depleted T cells and tumor‐infiltrated natural killer (NK) cells also showed dysregulation of mitochondrial biosynthesis. This has aroused the interest of researchers in promoting tumor immunogenicity through strategies that improve mitochondrial biosynthesis.^22^ In addition to the significant effect of hypoxia on tumor immunogenicity, researchers are actively exploring novel mechanisms of immune escape and resistance to ICBs, including other aspects of the TME influenced by metabolic conditions, such as changes in nutrient sources.^21^ Notably, the content and release of exosomes are affected by hypoxia.^23^ A variety of cells secrete exosomes, which are defined as nanoparticles with a lipid bilayer that show biological activity, which mediates signal communication between cells.^24^ Such interactions in the TME are vital for cancer progression. Evidence suggests that more aggressive cancer phenotypes are induced by hypoxia. Non‐coding RNAs (ncRNAs)shuttling through exosomes from hypoxic tumors have been shown to be fundamental molecules that regulate cancer biology and reshape the TME.^25^ In addition, exosomal ncRNAs from hypoxic tumors could be detected in body fluids as promising diagnostic and prognostic biomarkers.^26^

Moreover, analysis of human samples revealed differences in the composition of the tumor microbiota between responders and non‐responders in patients with melanoma receiving immunotherapy.^27^ Tumor‐associated microorganisms have been associated with reduced immune cell infiltration and remodeling of the immunosuppressive microenvironment. Intratumoral microbes, another component of the TME, were previously underestimated; however, they have been shown to impact tumor immune responses and ICB responses significantly.^28^ The characteristics of TME mainly fall into three categories: hypoxia, chronic inflammation, and immunosuppression. They complement each other and form a complex mechanistic network, which plays a key role in many steps of tumor development, such as local drug resistance, immune escape, and distant metastasis.

### 2.2 Cancer progression is driven by metabolic crosstalk in the TME

Research indicates that alteration to tumor metabolism leads to a sufficient supply of energy and the production of the metabolic intermediates for tumor growth, and helps to inhibit the anti‐tumor response. Frequently, TME immunosuppression is characterized by the mutual metabolic demands of tumor cells and immune cells. NK and cytotoxic T‐cell activation increase the demand for amino acids and glucose, which is characteristic of tumor cells.^29^ This metabolic interdependency results in competition for metabolites, which constrains the effector function and proliferation of tumor‐specific immune cells. Besides, changes in the abundances of metabolites and the buildup of metabolic waste products (e.g., lactic acid) driven by tumors, result in local immunosuppression, which in turn encourages tumor metastasis and progression.^30^

**FIGURE 1:**
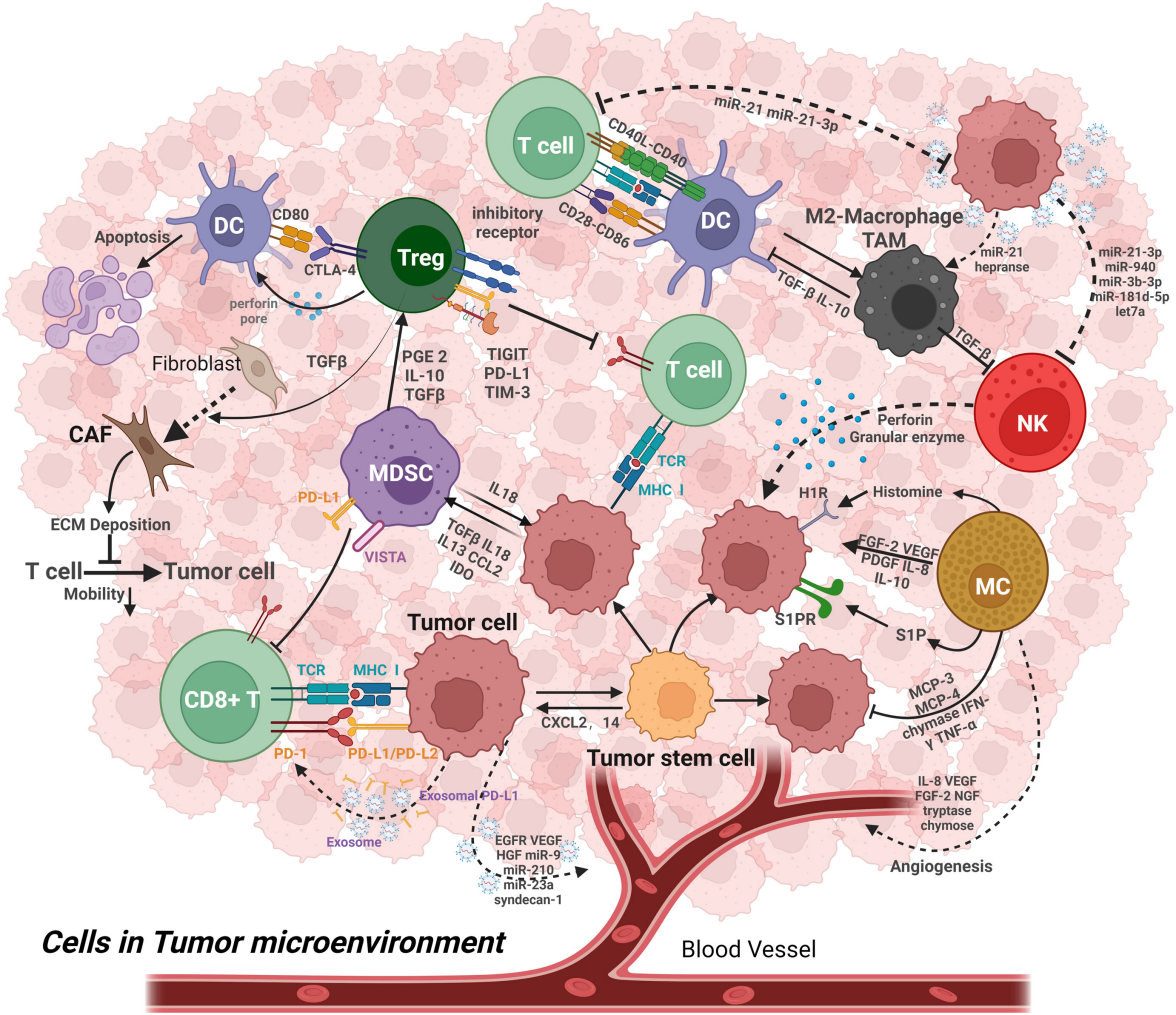
The cellular and structural components in the tumor microenvironment.

Metabolism strongly shapes the interaction between cancer cells and immune cells, modulating the anti‐tumor immune response (Figure 1). Most cancer cells are characterized by glycolysis, followed by lactic acid and TME acidification, which are associated with several aggressive parameters, including immune evasion.^31^ The first step in glycolysis, the conversion of glucose to glucose‐6‐phosphate, is catalyzed by hexokinases (HK). Among the four subtypes found in mammalian tissues, HK2 has been reported to be particularly overexpressed in cancer tissues.^32^ It has been shown that HK2 is required for tumorigenesis in an ErbB2/ Her2‐driven breast cancer mouse model, and HK2 ablation inhibits the malignant phenotype of breast cancer cells in vitro and in vivo.^33^ Furthermore, the third step of glycolysis, the conversion of phosphoenolpyruvate (PEP) to pyruvate, is catalyzed by pyruvate kinase (PK). PKM2 isoform is highly expressed in highly proliferative cells of several cancer types. In addition to playing a key role in glycolysis, PKM2 is also involved in tumorigenesis by acting as a co‐activator and protein kinase.^34^ The clinical significance of elevated PKM2 in breast cancer was recently mentioned in a meta‐analysis, where high expression of PKM2 predicted poor survival in breast cancer patients and was associated with lymph node metastasis.^35^

When tumor cells form pro‐tumorigenic cocoons, reprogramming occurs in the tumors and their constituent cells to exploit the interaction with neighboring cells to ensure a constant supply of anaplerotic molecules and nutrients to fuel tumor cell growth, even under conditions of hypoxia.^36^ In addition, tumor cell metabolic reprogramming is important to suppress immune attacks and therapeutic resistance. The IGF‐1R/mTOR pathway is the most common abnormal activation of the growth factor signaling cascade, and IGF‐1 regulates osteogenesis and homeostasis, which plays an important role in the pathogenesis of sarcoma.^37^, ^38^ However, the PI3K/Akt pathway downstream of growth factor receptor tyrosine kinase is dysregulated in many solid tumors by multiple mechanisms, including the IGF‐1 signaling pathway, which regulates a variety of cellular functions such as metabolism, cell survival, proliferation, and protein synthesis via PI3K/Akt/mTOR.^39^, ^40^ The IGF‐1R/mTOR pathway is frequently activated in sarcomas and other solid tumors, so it is considered an attractive target for Osteosarcoma (OS) and Ewing sarcoma (ES) since it represents the aggregation of multiple chemical and mechanical signaling mechanisms. In addition to intrinsic genetic and epigenetic changes to tumor cells, metabolic competition and cooperation among various TME constituents also support tumor cell growth, metastasis, and drug resistance.^41^ The above‐described metabolic complexity and plasticity represent a serious threat to our ability to develop drugs that target selective metabolic signatures of the TME.

There are many immune cells in the tumor microenvironment. The interaction between immune cells and tumor cells, and the interaction between immune cells and immune cells, are regulated by the tumor microenvironment and cell surface immune checkpoints. Tumor stem cells drive tumor growth and influence the intrinsic heterogeneity of tumors.^42^ Mast cells (MC) are recruited near tumors during tumorigenesis and release a variety of cytokines, chemokines, etc.^43^ Natural killer cells (NK) are cytotoxic and secrete tumor necrosis factor and perforin to kill tumor cells.^44^ Tumor‐associated macrophages (TAM) are macrophages that infiltrate around tumor cells and are mainly divided into two categories: classically activated macrophages (M1 type) and alternatively activated macrophages (M2 type), of which M2 type macrophages can promote immune escape from tumors.^5^ Dendritic cells (DC), as antigen‐presenting cells, can induce immune responses but are often dysfunctional and apoptosis in TME.^45^ Myeloid‐derived suppressor cells (MDSC) have immunosuppressive effects, can block immunotherapy, and play a role in tumor maintenance and progression.^46^ Regulatory T cells (Tregs), a subpopulation of T cells, also have a role in suppressing the immune system, Treg can downregulate T cell induction and proliferation, and large numbers of Treg in TME often predict a poor prognosis.^47^ Normal fibroblasts can be activated into cancer‐associated fibroblasts (CAFs) in TME, and CAFs can provide a favorable microenvironment for tumor cells, promoting tumor growth and metastasis.^48^

### 2.3 Other factors

The long‐term dynamic interactions between epithelial breast cancer cells and matrix components (lymph cells, endothelial cells, and fibroblasts) were modeled in three‐dimensional in vitro cultures,^49^ which showed images of the protein hydrolysis that occurs in the TME during tumor cell invasion. The TME has important functions in mesenchymal stem cell reprogramming and tumor stems cell characteristic maintenance. Angiogenesis is promoted by matrix factors, which support tumor growth and metastasis. In cancer development, the inflammatory response in the microenvironment and reactive oxygen species (ROS) production are important factors. Matrix‐produced NO activates pro‐cancer signaling pathways of estrogen receptor (ER)‐negative breast cancer cells and induces white blood cells to synthesize transforming growth factor beta (TGF‐β) and interleukin 10 (IL‐10), which have immunosuppressive effects^50^ and iNOS is a powerful predictive marker of poor prognosis.^50^

**FIGURE 2:**
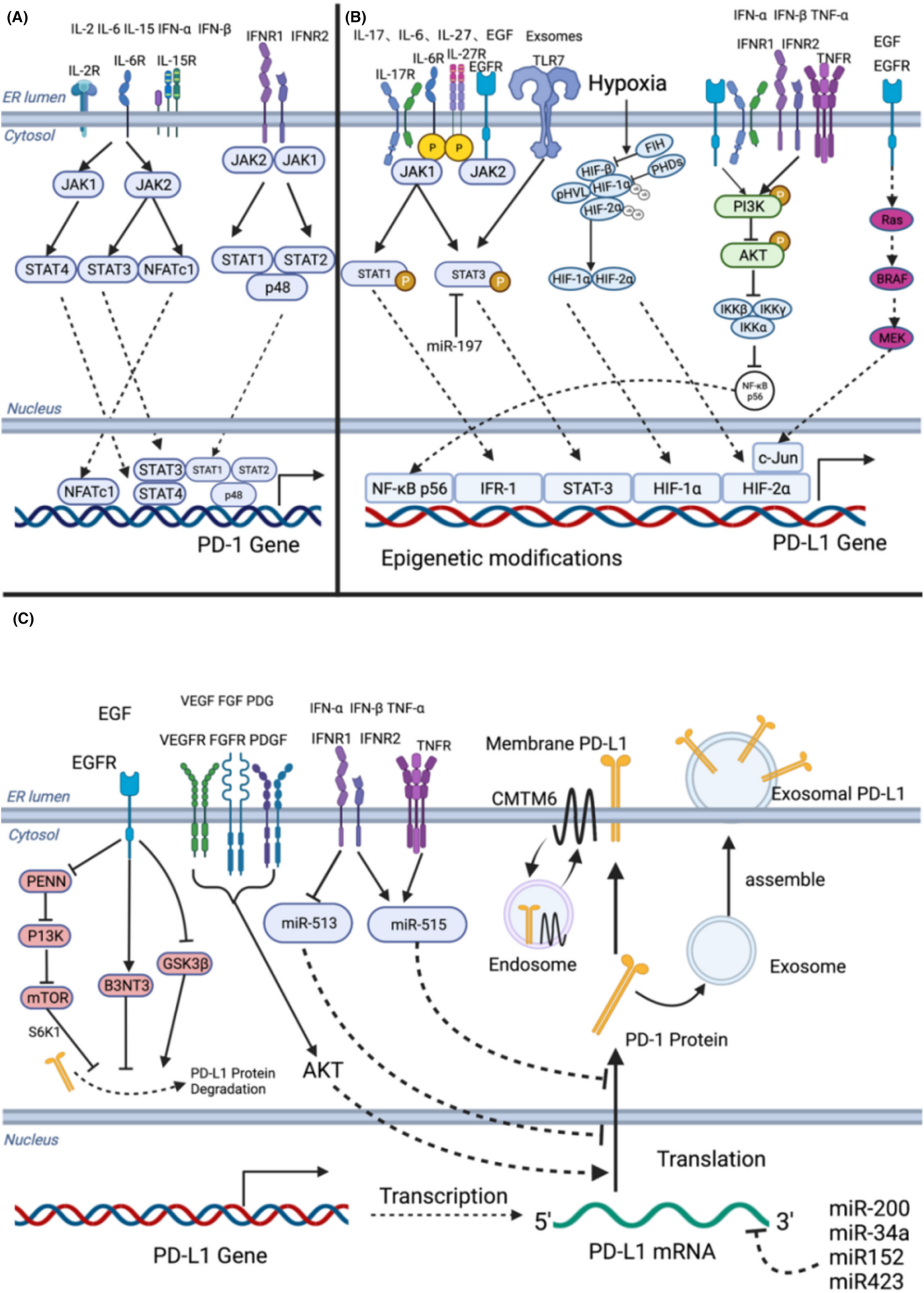
The regulatory network of PD‐1/PD‐L1 expression in the tumor microenvironment.

Potential oncogenic compounds might influence the TME to promote multiple stages of tumor development, by influencing epithelial cells (the most common cancer‐origin cells), immune cells, ECM components, and stromal cells. Therefore, further study of the dose‐dependent effects of chemicals and their mixtures on the TME could reveal important general mechanisms of tumor etiology and tumorigenesis prevention.^51^ Hypoxia, as one of the characteristics of TME, also induces PD‐L1 upregulation. Hypoxia‐induced factor 1 alpha (HIF‐1α) and nuclear factor kappa B (NF‐κB) are activated under hypoxic conditions.^52^ HIF‐1α can act on the hypoxia response element‐4 (HRE‐4) of the PD‐L1 proximal promoter, thereby improving PD‐L1 mRNA expression.^53^ Hypoxia can also induce cell damage, immune cell activation, and chronic inflammation, which releases TNF‐α And IFN‐γ other cytokines, which can induce cells to express PD‐L1.^52^ (Figure 2).

Cytokines, IL‐2, IL‐6, IL‐15, IFN‐α, and IFN‐β, via their corresponding receptors and signal pathways, induce PD1 expression. For example, IL‐6 improves PD1 transcription through the STAT3‐STAT4 pathway and during TCR activation and alters the PD1 chromatin structure. (B) Expression regulation and epigenetic modification of PDL1: Besides common cytokines, hypoxia factors in the TME also pass through overactivated HIF‐1α and NF‐κB to increase PDL1 expression, including HIF‐1α binding to its proximal promoter. (C) Translation of *PDL1* and PD‐L1 protein regulation: During PDL1 translation, small molecules such as miR‐200 and miR‐34a, can bind to one end of the PDL1 mRNA to inhibit transcription, PD‐L1 protein degradation is regulated by EGF, and VEGF and FGF also decrease or promote translation through corresponding signal pathways. The synthesized PD‐L1 is expressed on the membrane, and can also be secreted via exosomes. Tumor occurrence is regulated by an abnormal immune response and changes in homeostasis.^4^ In the TME, cancer cell, and immune cell interactions affect the growth and metastasis of cancer.^10^, ^16^

## 3 THE TME PLAYS AN IMPORTANT ROLE IN THE REGULATION OF PD‐1 AND PD‐L1 EXPRESSION

Tumors increase their chance of survival via the important strategy of tumor immune escape, which includes many mechanisms, among which immunosuppression has become a hot topic of research in recent years.^9^ Tumor‐induced immunosuppression has two main modes of action. First, immunosuppressive cells are induced to aggregate around the tumor and secrete immunosuppressive factors that lead to the inactivation of CTLs, such as regulatory T cells (Treg cells),^54^ Myeloid suppressor cells (MDSCs), dendritic cells (DCs), and M2‐type macrophages,^55^ thereby reducing the immune tolerance of tumor cells. Second, the expression of immunosuppressive molecules or their receptors are induced, including PD‐L1/PD‐1, galectin‐9‐T cell immunoglobulin mucin 3 (TIM3), indoleamine 2,3‐doxygenase 1 (IDO1), lymphocyte activating 3 (LAG3), and cytotoxic T‐lymphocyte associated protein 4 (CTLA4), termed immune checkpoints, which inhibit effector T lymphocyte activation, leading to immune escape of tumors. Therefore, in recent years, immunotherapy research has sought to block these immune checkpoints to reduce immunosuppression and restore immune system function.^54^

The Ig receptors PD‐L1 and PD‐L2 are expressed on the cell surface,^56^ and interact with PD‐1 to mediate tumor immune escape. PD‐L1 exists in both hematopoietic and non‐hematopoietic cells, and its expression is regulated by interferon‐gamma (IFN‐γ) and cytokines including IL‐7 and IL‐15.^17^, ^57^, ^58^ The PD‐1/PD‐L1 pathway can inhibit the activation, proliferation, and corresponding cytotoxicity of T cells in the TME.^9^ The expression of PD‐L1 is also closely related to the effect of anti‐PD‐1/PD‐L1 therapy on non‐small cell lung cancer, metastatic urethral cancer, melanoma, and other cancers.^59^ PD‐L2 is expressed on the surface of DCs, macrophages, mast cells, and some B cell groups. PD‐L2 is significantly expressed in the TME of renal cell carcinoma (RCC) and lung squamous cell carcinoma (LUSC).^60^ The simultaneous expression of PD‐L1 and PD‐L2 in tumor cells can lead to a significantly inhibited anti‐tumor immune response.^59^, ^60^ In the future, anti‐PD‐L2 monoclonal antibody applications might overcome the limitations of anti‐PD‐L1 monoclonal antibody drugs (Table 1).

**TABLE 1:** Monoclonal antibody drugs approved by the FDA for corresponding treatment regimens from 2020 to 2021.

Target	Antibody	Producer	tumor type	Response rate	NCT number
PD‐1	Pembrolizumab	Merck	TMB‐H solid tumors	TMB ≥10 mut/Mb: ORR = 29% (with 4% complete response rate and 25% partial response rate)	NCT02628067
			Cervical cancer	ORR = 68%, median DOR = 18.0 months, median PFS = 10.4 months	NCT03635567
			Cutaneous squamous cell carcinoma	ORR = 34%, median response duration was not reached	NCT03284424
	Nivolumab	Bristol‐Myers Squibb	Urothelial carcinoma	ITT analysis: median DFS = 20.8 months, Patients with tumor expressing PD‐L1 ≥ 1%: median DFS not reached	NCT02632409
			Esophageal GEJ cancer	Median DFS was 22.4 months	NCT02743494
			Esophageal squamous cell	Median OS = 10.9 months, ORR = 19.3%, median response duration = 6.9 months	NCT02569242
	Cemiplimab	Regeneron Pharmaceuticals	Non‐small cell lung cancer with high PD‐L1 expression (TPS ≥50%)	Median OS = 22.1 months, median PFS per BICR = 6.2 months, ORR = 37%	NCT03088540
			Locally advanced and metastatic basal cell carcinoma	Patients with laBCC: ORR = 29%, patients with mBCC: ORR = 21%	NCT03132636
	Dostarlimab‐gxly (Jemperli)	GlaxoSmithKline	dMMR advanced solid tumors	ORR = 41.6% (with 9.1% complete response rate and 32.5% partial response rate)	NCT02715284
			dMMR endometrial cancer	Median DOR = 34.7 months (95% of patients with duration ≥6 months), ORR = 42.3% (with 12.7% complete response rate and 29.6% partial rate)	NCT02715284
PD‐L1	Durvalumab	AstraZeneca	Extensive‐stage small cell lung cancer	Median OS = 13.0 months, median PFS = 5.1 months, ORR = 68%	NCT03043872
	Avelumab	EMD serono	Urothelial carcinoma	OS = 21.4 months, PD‐L1 positive tumors: HR for OS was 0.56, PD‐L1 negative tumors: OS HR was 0.85	NCT02603432
	Atezolizumab	Genentech	Metastatic NSCLC with high PD‐L1 expression	OS = 20.2 months, PFS = 8.1 months, ORR = 38%	NCT02409342

Abbreviations: BICR, blinded independent central review; DFS, disease‐free survival; DOR, duration of response; FDA, USA Food and Drug Administration; HR, hazard ratio; ITT, intention to treat; laBCC, locally advanced basal cell carcinoma; mBCC, metastatic basal cell carcinoma; ORR, overall response rate; OS, overall survival; PD‐1, programmed cell death‐1; PD‐L1, programmed cell death‐1 ligand‐1; PFS, progression‐free survival; TMB, tumor mutation burden.

There are complex molecular signaling networks in the TME, in which cytokines and growth factors affect related signal pathways and regulate PD‐1 and PD‐L1 expression.^9^, ^61^ The TME can increase IFN‐γ, TNFα, and IL‐6 to up‐regulate PD‐L1 expression. For example, IL‐6 can strengthen the connection with the n‐glycosyltransferase STT3 oligosaccharyltransferase complex catalytic subunit A (STT3A) and upregulates PD‐L1 expression via the IL‐6‐Janus kinase 1 (Jak1) pathway.^62^ In the TME, PDL1 mRNA translation can be inhibited by upregulating the expression level of miR‐155,^63^ or miR‐155‐related translation inhibition can be weakened by inhibiting the expression of miR‐155, thereby enhancing PD‐L1 protein levels.^64^

In addition, various cells in the TME secrete bioactive lipid bilayer nanovesicles termed exosomes,^24^ which usually contain a variety of RNA, DNA, proteins, miRNAs, and other bioactive molecules.^65^ PD‐L1 can transfer to other tumor cells through exosomes, induce drug resistance of receptor cells, and promote tumor development and metastasis.^66^ For example, HER2 cells can release exosomes containing PD‐L1, which enables cells sensitive to HER2‐targeted drugs to acquire drug resistance.^67^

## 4 RECENT DEVELOPMENTS IN TUMOR IMMUNOTHERAPY

### 4.1 Targeting monoclonal antibodies and combination therapies

Maintenance of immune homeostasis by inhibiting T cell activation is achieved by the binding of PD‐L1 to PD‐1. Tumor cells express high levels of PD‐L1, which bind to PD‐1 on activated T cells, resulting in tumor immune escape. The binding of PD‐1 to PD‐L1 can be prevented using ICBs such as anti‐PD‐1/PD‐L1 antibodies, which rejuvenate depleted T cells and thus inhibits tumor growth. Tumor cells promote tumor EMT, angiogenesis, and immune escape via exosomes, which carry PD‐L1 with the same membrane topology as that on the cell surface, thus resisting ICB therapy.^24^ Even in models resistant to PD‐L1 antibodies, the removal of exosomal PD‐L1 inhibits tumor growth. Tumor exosomal PD‐L1 inhibits T cell activation in draining lymph nodes. Systemically‐introduced PD‐L1 exosomes rescued the growth of tumors that could not secrete their own hormones. Anti‐PD‐L1 antibodies complemented rather than replaced exosome PD‐L1 blockade to inhibit tumor growth. Thus, exosomal PD‐L1 represents a novel therapeutic target that could bypass resistance to current antibodies.^68^

Since May 2006, the FDA has successively approved three anti‐PD‐L1 drugs (atezolizumab, avlumab, and durvalumab) and four anti‐PD‐1 drugs (nivolumab, pembrolizumab, cemiplimab, and dostarlimab‐gxly). As an IgG1 monoclonal antibody (mAb), atezolizumab has the characteristics of high affinity and humanization. It can combine with PD‐L1, prevent it from interacting with PD‐1 and B7‐1 (the CD80 receptor), and improve the immune activity of T cells against tumors.^69^ Atezolizumab has been approved for the treatment of urethral cancer, non‐small cell lung cancer, locally unresectable or metastatic triple‐negative breast cancer (TNBC), and other diseases.^70^ Avlumab is a humanized IgG1 mAb that has been approved to treat Merkel cell carcinoma, locally advanced or metastatic bladder cancer, and other diseases.^70^, ^71^ It is characterized by a dual action mechanism. It not only inhibits the interaction between PD‐L1 and PD‐1, thus reducing immunosuppression in the TME, restoring effector T cell activity and antitumor activity, but also mediates an antibody‐dependent cell‐mediated cytotoxicity (ADCC) effect through its primary FCR fragment to make NK cells kill tumors.^72^ Durvalumab is an IgG1κ1 mAb that binds PD‐L1, with the characteristics of high selectivity and high affinity,^70^, ^73^ which has been approved for the treatment of non‐small cell lung cancer, urethral cancer, and extensive small cell lung cancer.

Nivolumab is a fully humanized IgG4 anti‐PD‐1 mAb that can prevent the binding between PD‐1 and its ligands PD‐L1 and PD‐L2, thus promoting T cell proliferation and cytokine production, and has a high affinity for PD‐1.^74^, ^75^ It has been approved to treat renal cell carcinoma, urothelial carcinoma, and lung cancer.^70^ Pembrolizumab is a humanized anti‐PD‐1 monoclonal IgG4‐κ alloantibody that can combine with PD‐1 to restore the anti‐tumor immune response and achieve an anti‐tumor effect.^70^, ^76^ Cemiplimab is a fully humanized IgG4 mAb that can bind PD‐1 to prevent its reaction with PD‐L1, thereby upregulating cytotoxic T cells and enhancing the immune system's antitumor activity. Cemiplimab has been approved for metastatic skin squamous cell carcinoma^70^ and is considered the first and only treatment for advanced skin squamous cell carcinoma.^77^ On April 22, 2021, the U.S. Food and Drug Administration (FDA) approved dostarlimab ‐Gxly for the first time as a single‐agent therapy for patients with recurrent or advanced endometrial cancer, misalignment repair defect (dMMR), or disease progression during or after platinum‐containing chemotherapy. Dostarlimab is a humanized anti‐PD‐1 monoclonal antibody that binds to the PD‐1 receptor and inhibits its interaction with the ligands PD‐L1 and PD‐L2.^78^ In the clinic, mAbs, as commonly used drugs, show long‐lasting anti‐tumor responses and high therapeutic effects.^13^, ^79^, ^80^ However, mAbs cannot be administered orally, and have a high molecular weight and high affinity, resulting in poor penetration of solid tumors, a low pharmacokinetic effect, and immunogenicity after long‐term administration, resulting in immune‐related adverse events (IEAEs). They also have disadvantage of high production costs.^13^, ^79^ The TME can promote matrix proliferation to reduce drug permeability.^81^ Tumor‐derived exosomes (TDE) in the TME contain a variety of internal and surface proteins, which participate in the formation and development of different cancers and mediate cancer resistance.^24^ For example, drug‐resistant cancer cells can wrap anticancer drugs in exosomes and transport them away from cancer cells.^82^ In addition, secretory PD‐L1 can resist PD‐L1 blocking therapy in non‐small cell cancer therapy and decrease the effect of mAb drugs.^83^

In view of the shortcomings of mAbs and the impact of the TME, combination therapy (Table 2) was developed, among which radiotherapy combined with ICB is the most common. The combined blockade of CTLA‐4 and PD‐1 has achieved good results in cancers, including renal cell carcinoma and melanoma.^84^ Combined treatment with nivolumab and ipilimumab significantly improved the progression‐free survival (PFS) rate of previously untreated patients with metastatic melanoma in the third phase of the study, The combination of the two drugs also reflects the therapeutic complementarity for patients with PD‐L1 negative tumors.^85^ Clinical trials also showed that anti‐PD‐L1 antibodies combined with radiotherapy promoted the penetration of CD8^+^ T cells and enhanced the antitumor effect on NSCLC synergistically.^86^ Combined therapy based on other immune checkpoints (such as B and T lymphocyte associated (BTLA)), T cell immunoreceptor with Ig and ITIM domains (TIGIT), TIM3, and LAG3 and multi‐stage drug delivery system (MS DDS) based on specific factors in the TME (such as hypoxia and pH) will also play an important role in future treatment.^30^, ^87^, ^88^

**TABLE 2:** Combination therapy with monoclonal antibodies.

Therapy and targets	Tumor type	Response rate	NCT number
Nivolumab(Anti PD‐1), chemotherapy	Metastatic cancer and esophageal adenocarcinoma	Median OS^†^ = 14.4 months, median PFS = 7.7 months	NCT02872116
Nivolumab (Anti PD‐1), ipilimumab (Anti CTLA‐4)	Hepatocellular carcinoma	ORR = 35%, responses duration ranged from 4.6 to 30.5 months with 31% of responses lasting at least 24 months	NCT01656878
Nivolumab (Anti PD‐1), ipilimumab (Anti CTLA‐4)	First‐line mNSCLC (PD‐L1 tumor expression ≥1%)	Median OS = 17.1 months, median PFS = 5.1 months	NCT02477826
Nivolumab (Anti PD‐1), ipilimumab (Anti CTLA‐4), chemotherapy	Metastatic NSCLC	Median OS = 14.1 months, median PFS = 6.8 months, ORR = 38%, median response duration = 10 months	NCT03215706
Atezolizumab (Anti PD‐L1), cobimetinib (Anti MEK), vemurafenib (Anti B‐Raf enzyme)	BRAF V600 unresectable or metastatic melanoma	Median PFS = 15.1 months	NCT02908672
Pembrolizumab (Anti PD‐1), chemotherapy	High‐risk early‐stage triple‐negative breast cancer (TNBC)	Pathological complete response (qCR) rate = 63%, EFS = 16%	NCT03036488
Pembrolizumab (Anti PD‐1), lenvatinib (Anti VEGFR1, 2 and 3 kinases)	Advanced endometrial carcinoma	Median PFS = 6.6 months, median OS = 17.4 months, ORR = 30%, median DOR = 9.2 months	NCT03517449
Pembrolizumab (Anti PD‐1), lenvatinib (Anti VEGFR1, 2 and 3 kinases)	Advanced renal cell carcinoma (RCC)	Median PFS = 23.9, ORR = 71%	NCT02811861
Pembrolizumab (Anti PD‐1), platinum, and fluoropyrimidine‐based chemotherapy	Esophageal or GEJ carcinoma	Median OS = 11.4 months, median PFS = 6.3 months	NCT03189719
Pertuzumab (Anti HER2), trastuzumab (Anti HER2/neu), nyaluronidose‐zzxf	HER2‐positive breast cancer	Pathological complete response (qCR) = 59.7%	NCT03493854

Abbreviations: BRAF V600, B‐raf mutation at valine 600; DOR, duration of response; EFS, event‐free survival; GEJ, gastroesophageal junction; HER2, human epidermal growth factor receptor; mNSCLC, metastatic non‐small cell lung cancer; ORR, overall response rate; OS, overall survival; PD‐1, programmed cell death‐1; PFS, progression‐free survival.

### 4.2 Radiotherapy affects the expression of PD‐L1 in the TME

Radiotherapy (RT), as a major anticancer method, can reorganize the TME, regulate the immune system,^89^, ^90^ and change the relationship between tumor cells and immune cells.^89^, ^91^ RT can induce cancer cell apoptosis,^92^ trigger immunogenic cell death (ICD), and stimulate the immune response through the interaction between pattern recognition receptors (PRRs) and damage‐related molecular patterns (DAMPs).^93^, ^94^ DAMPs can be divided into three categories: surface‐exposed calreticulin (CRT), secreted ATP, and released high mobility group protein B1 (HMGB1).^93^, ^94^ CRT can increase the number of phagocytes, mediated by CD91; HGMB1 can activate Toll‐like receptor (TLR)2 and 4 to activate the production of DCs and inflammatory cytokines through myeloid differentiation primary response 88 (MyD88) signaling, and ATP can activate purinergic receptor P2X7 (P2X7) and purinergic receptor P2Y2 (P2Y2) and stimulate NK cells, T cells, macrophages, and DCs.^94^ Radiation can activate the NF‐κB pathway, and induce TNFα, IL‐6, IL‐1α, and IL‐1β. The expression of other proinflammatory cytokines increases tumor inflammation.^95^ Radiation leads to cell apoptosis and releases multiple radiation‐related antigens (RAAPs).^89^ The release of RAAPs and the inflammatory response can promote the development and maturation of DCs and stimulate tumor‐specific T cells.^95^ ATP released from cancer cell death induced by RT is rapidly decomposed into adenosine in the TME by CD39 and CD73 expressed on immune cells, such as regulatory T cells (Tregs) and T helper 17 cells (Th17), tumor cells, and stromal cells.^96^ The accumulation of adenosine will increase the expression of CTLA4 and adenosine receptor 2A (A2AR) on Tregs, promote Treg proliferation and enhance tumor‐associated macrophage (TAM) differentiation into the M2 inhibitory phenotype. The accumulation of adenosine also inhibits DCs and effector T cells and weakens the immune response.^97^, ^98^ In addition, radiation affects the expression of PD‐L1 in tumor tissues, such as chemical radiation increased the expression of PD‐L1 in some glioblastoma and melanoma cells.^99^ Recently, using neoadjuvant chemotherapy (NAC) to treat patients with cervical cancer, researchers found that the proportion of patients with high expression of PD‐L1 increased significantly from 32.4% to 46.5%, indicating that chemotherapy can PD‐L1 expression in cervical cancer.^100^ RT can increase or weaken the immune response in many forms of cancer. The radiation‐induced tumor equilibrium (RITE) and radiation‐induced distal effect^101^ are still important factors that need to be considered; however, further research is required.

### 4.3 Peptides and macrocyclic inhibitors

Small molecules generally refer to organic compounds with a molecular weight of less than 900 daltons, and most drugs today are small molecule drugs that act as inhibitors to interfere with protein interactions. Anticancer therapy based on ICB has made amazing achievements in the past few years.^102^ Recently, small molecule inhibitor therapy has been used actively in research and clinical experiments because of its specificity, high affinity, low immunogenicity, good pharmacokinetics, low cost, and easy transportation.^102^, ^103^ Many specific peptides can also be used as the objects of vaccine research and development. For example, peptides that mimic PD‐1 epitopes can be used in vaccination.^79^ The 29‐mer peptide, aunp‐12, developed jointly by Pierre Fabre and Aurigene Discovery Technologies Limited, can target PD‐1/PD‐L1 immune checkpoints, and could inhibit the growth of melanoma cells by 44% in a b6f0 mouse model.^80^ Researchers have developed synthetic peptides blr100 and blr200 from the active region of cellular communication network factor 3 (CCN3). These two peptides are characterized by inhibiting CCN3. Experiments have found that blr100 and blr200 could reorganize the TME, affect the activity of fibroblasts, inhibit fibrosis and angiogenesis, and reduce the necrotic region of the tumor.^104^ Peptidomimetic 7, a peptide developed by Aurigene, was applied in a CT‐26 colon cancer mouse model. The results showed that the peptide could inhibit 46% of tumor growth.^80^ Researchers also constructed a PD‐1 binding peptide showing that the use of the binding peptide inhibited the receptor signal of PD‐1. In a B16‐F10 mouse melanoma model, it was found that the combination of peptides sq20, qp20, WQ20, and HD20 and an anti‐PD‐1 mAb greatly reduced tumor metastasis.^105^ Bristol Myers Squibb (BMS) has developed two macrocyclic peptides, bms‐57, and bms‐71. They verified that these two peptides have an affinity for PD‐L1, are similar to antibodies, can inhibit PD‐L1 signaling, and restore T cell function.^102^ In addition to synthetic small molecular peptides, small molecular substances obtained from plants might represent new inhibitors. Curcumin extracted from plant Turmeric can recognize and inhibit the interaction between tumor cells and stromal cells, interfere with the synergy between colorectal cancer and tumor stem cells and fibroblasts in the high‐density tumor microenvironment, improve the sensitivity of colorectal cancer hepatocytes to chemotherapeutic drugs, and directly affect colorectal cancer stem cells.^13^ The research and development of small molecule inhibitors are expected to overcome the shortcomings of certain mAbs, and the research and development of related oral inhibitors are also advancing, which is expected to become a strong strategy to control the condition of patients with cancer.

### 4.4 Genetically engineered cell therapy and TME

Chimeric antigen receptor T cells are a type of T cell^106^ that has been genetically engineered to express a specific antigen receptor. As a modular fusion protein, the CAR is usually composed of an extracellular targeted binding domain, a spacer domain, a transmembrane domain, and an intracellular signal domain derived from an antibody single chain variable fragment (scFv).^107^ A CAR is usually encoded by an adenovirus, retrovirus, lentivirus, and other viral vectors and plasmids, which are introduced into T cells, among which lentivirus has become the most common transduction method of human T cells.^106^ Since 2017, the FDA has successively approved kymriah, yescarta, tecartus, and Liso‐cel, which are four CAR‐T products targeting B cell CD19, and also approved abecma, the first CAR‐T product targeting B cell maturation antigen (BCMA) in 2021. CAR‐T treatment has achieved great success in B‐cell acute lymphoblastic leukemia (B‐ALL) or B‐cell malignancies, in which the complete remission rate for B‐ALL can be as high as 90%.^107^ However, the effect of CAR‐T in the immunotherapy of solid tumors is still poor. The main factors are the immunosuppressive TME^108^ and the antigen heterogeneity of solid tumors.^109^ The activity and persistence of CAR‐T itself are also important factors affecting treatment. Although antigen escape mostly occurs in hematological tumors, it has also been reported in solid tumors, for example, non‐targeted cells expressing a tumor‐associated antigen (TAA) appear in the treatment of glioblastoma.^110^

Hypoxia, acidity, nutrient deficiency, and other factors in the TME can cause the proliferation and dysfunction of CAR‐T cells and stimulate oxidative stress.^111^ The physical barrier constructed by it, such as VEGF‐induced angiogenesis, the proliferation of tumor‐related fibroblasts, the deposition of the ECM, and the decrease of cell mobility, will prevent CAR‐Ts from contacting tumor cells and playing their role.^106^ Soluble factors from the tumor, such as prostaglandin E2 (PGE2) produced by tumor cells and macrophages, and adenosine produced at high levels during hypoxia, can signal through their own G‐coupled receptors to activate protein kinase A (PKA) to induce immunosuppression. Cytokines in the TME, such as TGF‐β, will also have a direct negative impact on the CAR‐T cell effect.^112^ In the TME, all kinds of inhibitory immune cells, such as Tregs, myeloid‐derived suppressor cells, tumor‐associated macrophages, and tumor‐associated neutrophils, are considered to have anti‐tumor immune effects; however, their effects on CAR‐T cells have not been widely studied.^109^ In addition, the TME can also upregulate the expression of immunosuppressive receptors, such as PD‐1 and CTLA‐4, on the surface of CAR‐T cells, and inhibit T cells through immune checkpoints.^109^ Considering the impact of the TME on CAR‐T cells, combined treatment with CAR‐Ts is becoming more and more important. Experiments have proven the enhanced effect of immune checkpoint blockade on treatment. In an experiment studying human CAR‐T cells with an immune‐deficient animal tumor model, it was found that PD‐1 blocking of anti‐human antibodies enhances the anti‐tumor effect of human cortin‐oriented CAR‐Ts.^113^ For the cytokine network in the TME, IL‐12, and IL‐18 are also available.^108^

In addition, CAR‐T therapy itself may bring neurotoxicity, cytokine release syndrome (CRS), and extra tumor cross‐reaction,^114^ causing damage to the body. For tumor cells with low antigen specificity, double‐targeted CAR‐T cells, such as T cells containing two CAR molecules and a CAR molecule containing two domains (TANCAR), have also been actively developed in the clinic.^115^, ^116^ Some scholars also used a genetic modification of cytokines^117^ and CRISPR‐cas9 technology to knock out TCR, HLA, PD‐1, or CTLA‐4 genes, thereby promoting the therapeutic effect of CAR‐Ts.^118^, ^119^

### 4.5 Cancer and tumor vaccines

With the development of cancer immunotherapy, cancer vaccine technology has also been developed. Cancer vaccines can be divided into two types: preventive and therapeutic vaccines. The preventive vaccine can induce immune memory to reduce the incidence rate of specific cancers, for example, human papillomavirus (HPV) and hepatitis B virus (HBV) vaccines. Therapeutic vaccines can activate and strengthen the body's immune system and control cancer.^120^ According to the composition of the vaccine, it can be divided into a cell vaccine, a nucleic acid vaccine, and protein or peptide vaccine composed of tumor or immune cell proteins and peptides.^120^ Researchers constructed tumor models of CT26 colon cancer and ID8‐VEGF ovarian cancer mice, prepared a gvax vaccine composed of irradiated tumor cells expressing granulocyte‐macrophage colony‐stimulating factor, combined the dual block therapy of PD‐1 and CTLA‐4 with the vaccine, and observed the activation of tumor‐infiltrating lymphocytes in mice, the production of antigen‐specific inflammatory cytokines and the decrease of regulatory T cells.^121^ Through anti‐PD‐1 therapy and combined injection of a cell vaccine based on the combination of DC cells with C‐C motif chemokine ligand 21 (CCL21) and tumor antigen, it was found that the combined therapy could increase the activity of DCs and tumo C‐C motif chemokine ligand 21r infiltrating T cells in the TME and induced mice to produce immune memory in the tumor mouse model induced by K‐RasG12Dp53null.^122^ The therapeutic DNA vaccine constructed by a DNA vector encoding a cancer‐specific epitope also played a synergistic role in the process of controlling MC38 tumor growth in combination with anti‐PD‐1 therapy.^123^ Protein and peptide vaccines have also shown positive results in tumor therapy. Recent studies have found that small molecular peptides can bind to cancer antigens to effectively induce an antitumor immune response.^79^ A new PD‐1 B‐cell peptide epitope vaccine (amino acids 92–110; PD1 vaxx) technology has also been developed. PD1 vaxx can prevent the transmission of the PD‐1 signal and induces the body to produce corresponding antibodies, causing an anti‐cancer effect similar to that of nivolumab. PD1 vaxx can also combine with an HER‐2 peptide vaccine (b‐vaxx) to strengthen the inhibition of colon cancer growth.^124^ The combination therapy of therapeutic HPV protein vaccine inoculated into the tumor combined with PD‐1 blocking also had synergistic effects and induced tumor regression.^125^ Tumor vaccines, especially therapeutic tumor vaccines, are gradually becoming a favorable auxiliary tool and play an important role in cancer immunotherapy.

### 4.6 Intestinal microbiota and cancer immunotherapy

The intestinal microbiota comprises a large number of microorganisms. Increasing evidence shows that intestinal microorganisms are closely related to cancer. For example, colorectal cancer (CRC) occurrence, progression, and metastasis are majorly affected by the intestinal microbiota,^126^ intestinal flora may play an important key role in hepatocellular carcinoma (HCC) patients treated with anti‐PD‐1 immunotherapy.^127^ The early detection of cancer and its screening and prognosis can be performed using changes to the intestinal microbiota as biomarkers,^126^, ^128^ and the intestinal microbiota can be used as a therapeutic target.^128^ Fecal bacterial transplantation (FMT), based on microbiota research, can also reduce the toxicity of immune checkpoint inhibitor (ICI) treatment.^129^ After FMT, researchers found that the microbiota could affect the IL‐12‐dependent Th1 immune response and promote tumor control in mice and patients during CTLA‐4 checkpoint‐blocking treatment, and protect intestinal function.^130^ FMT and anti‐PD‐1 alter the gut microbiome and reprogram the tumor microenvironment to overcome resistance to PD‐1 in a subpopulation of advanced melanoma.^131^ In‐human clinical trials, FMT therapy has been associated with favorable changes in immune cell infiltration and gene expression profiles in the intestinal lamina propria and tumor microenvironment.^132^ In addition, the effect of the T‐cell response and tumor control was strengthened, and the effect of anti‐PD‐L1 treatment was improved using FMT.^133^ In a recent study, A strain engineered to express the tail length tape measure protein (TMP) epitope improved immunotherapy in mice. In patients with kidney and lung cancer, the presence of enterococcal phages in feces and expression of TMP cross‐reaction antigen in tumors are associated with long‐term benefits of PD‐1 blocking therapy.^134^ Furthermore, the concentration of L‐arginine in tumors was increased by developing a non‐pathogenic engineered *E. coli* that can colonize tumors and use the tumor metabolite ammonia, playing a synergistic role against PD‐L1 treatment,^135^ and maintaining healthy gut flora can help patients combat cancer.^127^, ^136^, ^137^ In the near future, the utilization of the intestinal microbiota might become a powerful tool for adjuvant cancer immunotherapy.

## 5 DISCUSSION—FUTURE PERSPECTIVES

Cancer was originally thought to be a disorder of cell and gene expression, whereas it is now also considered a TME disease. Characteristics of the TME can identify new prognostic and predictive biomarkers, leading to the identification of novel targets for therapy and their associated strategies, potentially guiding algorithms to determine first‐line therapy. Although the TME drivers of an individual's primary lesion site vary, many features are the same between patients. The detection of an anti‐tumor environment, characterized by a large number of Th1 cells, CD8^+^ T cells, and their related cytokines usually show that the immune system has contained the tumor to some extent, and can even lead to tumor elimination. Over the past 20 years, significant progress has been made in understanding the complexity of the TME and its impact on responses to various anti‐cancer therapies, especially immunotherapy.^30^ PD‐1 and CTLA‐4‐targeting immunotherapies have achieved notable clinical success, prompting increased research to gain a better understanding of tumor immunity. Other receptors, LAG3 (a negative regulator of CD4^+^ T cell activation), Nectin family (consists of the activating receptor CD226 and its major negative regulatory counterpart T cell immune receptor), and activated receptors on T cells such as CD137 agonist antibodies, TNFRSF4, GITR (also known as TNFRSF18), CD27 (also known as TNFRSF7), and ICOS show excellent activity. However, agonists have not shown significant clinical activity in humans. The reasons for the lack of efficacy include insufficient agonist activity, insufficient exploration of the dosage and protocol required for activation, down‐regulation of target receptors, and over‐activation that may lead to T cell apoptosis.^138^ The tumor immune environment affects the response to treatment and the survival of patients with cancer. It is imperative to identify treatments that can reduce immunosuppression, alleviate T cell failure, and enhance the TME effector function. In particular, it is critical to develop combination therapies for the TME that decrease immunosuppressive immune cell accumulation (e.g., TAMs and FOXP3+ Tregs) while promoting the activities of CD4+ and CD8^+^ effector T cells.^139^ Such treatments could effectively reshape the TME and promote the cancer immune response.

Immune checkpoint blockade therapy fails to induce a response in most cancer patients; therefore, how to improve the objective response rate has become an urgent challenge. Tumors are targeted with LIGHT (also known as TNF Superfamily Member 14 (TNSF14)), a member of the tumor necrosis factor superfamily, to activate lymphocytic toxin receptor signals, leading to the production of chemokines, and the recruitment of large numbers of T cells. In addition, antibody‐guided LIGHT targets non‐T‐cell‐inflamed tumor tissue, creating a T‐cell inflammatory microenvironment and overcoming tumor resistance to checkpoint blockade. Data suggest that targeting LIGHT might be an effective strategy to increase the response of non‐T‐cell inflammatory tumors to checkpoint blockade and other immunotherapies.^140^ In the future, the use of multiplex and multimodal biomarkers to characterize the host's anti‐tumor immune response might help to predict which patients will respond to immune‐based treatments. Research has identified potential therapeutic targets in immune microenvironments, thus ICIs that target the PD‐1/PD‐L1 axis have shown great promise. The FDA has approved anti‐PD‐1/PD‐L1 treatments for many types of tumors, some of which include PD‐L1 immunohistochemical diagnosis. Another unexplored question in the field is how specific carcinogenic drivers in tumor cells promote and shape the TME, and how this leads to the diversity of stromal cells, often in the same tissues.^10^ Other challenges include identifying which patients would benefit from immunotherapy, which combinations of anti‐cancer therapies and TME‐targeted drugs are the most effective, and how to ameliorate or bypass intrinsic or acquired resistance in the TME. These challenges are complicated by the TME's ability to affect tumorigenesis both favorably and unfavorably. In addition, the TME can normalize tumor cells, which implies that stromal cell re‐education, rather than targeted tumor ablation, could represent an efficacious modality to treat cancer.^141^, ^142^ In the future, dysfunctional TME re‐education might have a significant benefit to control and mitigate cancer, which is supported by the notable success of cancer immunotherapy reported so far.

The TME is one of the main features of cancer; therefore, identifying key drug‐acting factors and pathways in the TME to improve the efficacy of current cancer treatments is a major challenge. A recent study by pharmaceutical companies stated that the average research and development cost of a successful treatment was $648 million (USD); however, only 13.8% of drugs in clinical trials finally obtain FDA approval. Oncology drugs proved to have a lower success rate, at around 3.4%. Early drug trial failure rates were high, partly because current in vitro models lack translatability.^143^, ^144^ Single‐layer models have proven to be inadequate; therefore, researchers started to model tumors using a three‐dimensional (3D) microenvironment.^145^, ^146^, ^147^ Such 3D‐engineered tumor models have provided data related to cellular phenotypes, disease progression mechanisms, and therapeutic response in sarcoma, especially physical and chemical cues in TME. TME biological signals such as the tumor vascular system and the interstitial have been assessed; however, fewer studies of infiltrating immune cells in tumor engineering models have been carried out.^36^ Metabolic complexity and plasticity represent a major challenge to targeting metabolic signatures in the TME.^148^, ^149^, ^150^ In the future, the metabolic flux should be quantified to determine the mechanism of cancer‐related metabolic reprogramming. Such metabolic flux can be assessed at the genomic scale using constraint or kinetic modeling. In constraint‐based models, steady‐state metabolic flux is modeled using a set of linear constraints.^151^ Meanwhile, dynamic (kinetic) models can assess the steady‐state values and transient behavior of cell fluxes and concentrations.^152^ Cell type or tissue‐specific metabolic characteristics can be reflected using context‐specific models constructed by integrating cell or tissue‐specific data. Existing modeling frameworks have a limited capacity to model the metabolic communication between immune cells and tumors in the TME, thus future developments might involve new mixed kinetic/stoichiometry formulations.

## 6 CONCLUSIONS

Tumor microenvironment is a new field facing great challenges and opportunities. A comprehensive understanding of TME and its role in tumor immune development and progression will provide a conceptual change for the study of the tumor‐immune‐precision medicine relationship. TME influences the response to treatment and survival benefits of cancer patients. Determining therapeutic modalities, to limit immunosuppression, alleviate T cell failure, and enhance effector function plays an important role in the TME. In particular, it is critical to limit the accumulation of tumor microenvironment via re‐educating the immunosuppressive immune cells of TME. In summary, this review provides an understanding of the relationship between TME and immunotherapy that can effectively reinvigorate the immune response against tumors via shaping the tumor microenvironment.

## AUTHOR CONTRIBUTIONS

**Qingjing Wang:** Conceptualization (equal). **Xueting Shao:** Methodology (equal); writing – original draft (equal). **Yuxuan Zhang:** Project administration (equal); software (equal); validation (equal); visualization (equal); writing – original draft (equal); writing – review and editing (equal). **Miaojin Zhu:** Formal analysis (equal); validation (equal). **Frederick X.C. Wang:** Methodology (equal); validation (equal). **Jianjian Mu:** Methodology (equal); visualization (equal). **Jiaxuan Li:** Formal analysis (equal); software (equal). **Hangping Yao:** Funding acquisition (equal); project administration (equal); resources (equal); supervision (equal). **Keda Chen:** Conceptualization (equal); funding acquisition (equal); project administration (equal); resources (equal); software (equal); supervision (equal); validation (equal); visualization (equal); writing – original draft (equal); writing – review and editing (equal).

## FUNDING INFORMATION

The work was supported by the National Natural Science Foundation of China grant #81872883 (to HPY).

## CONFLICT OF INTEREST STATEMENT

The authors declare that they have no competing interests.

## Data Availability

Data sharing is not applicable to this article as no new data were created or analyzed in this study.
